# Antimicrobial mechanisms due to hyperpolarisation induced by nanoporous Au

**DOI:** 10.1038/s41598-018-22261-5

**Published:** 2018-03-01

**Authors:** Naoki Miyazawa, Masataka Hakamada, Mamoru Mabuchi

**Affiliations:** Graduate School of Energy Science, Kyoto University, Yoshidahonmachi, Sakyo, Kyoto, 606–8501 Japan

## Abstract

Nanomaterials such as nanoparticles exhibit remarkable antimicrobial activities. Nanoparticles directly disturb the cell membrane or cytoplasmic proteins because they pass through the cell wall. Nanoporous Au (NPG) is another antimicrobial nanomaterial, which cannot pass through the cell wall of bacteria but can still kill bacteria, utilising interactions between the surface of NPG and cell wall of bacteria. The origins of antimicrobial activities without direct interactions are unknown. It is necessary to elucidate these mechanisms to ensure safe usage. Here we show that the antimicrobial mechanism of NPG consists of two interactions: between the surface of NPG and cell wall, and between the cell wall and cell membrane. Fluorescent experiments showed that the cell wall was negatively hyperpolarised by NPG, and molecular dynamics simulations and first-principles calculations suggested that the hyperpolarisation of the cell wall leads to delicate structural changes in the membrane proteins, rendering them bactericidal. Thus, the hyperpolarisation induced by NPG plays a critical role in both interactions. The combination of molecular dynamics simulations and first-principles calculations allows a deeper understanding of the interactions between metallic surfaces and biomolecules, because charge transfer and exchange interactions are calculated exactly.

## Introduction

Bacteria such as *Escherichia coli* (*E*. *coli*) and *Staphylococcus epidermidis* (*S*. *epidermidis*) sometimes cause harmful effects to humans: for example, *E*. *coli* behaves as an etiologic agent when it intrudes into blood vessels or the urinary system. Many nanomaterials, such as metal-free photocatalysts^1^, Ag and Cu nanoparticles^2–6^ and so on^7–9^, show a remarkable ability to kill bacteria by the release of diverse antimicrobial species, such as reactive oxygen species (ROS) and metallic ions. Some surface-modified nanoparticles exhibit prominent antimicrobial activities without the release of harmful diffusive species^10–13^, although some surface-modified nanoparticles show antimicrobial activities with the release of ROS^14–16^. In the case of the surface-modified nanoparticles without the release of harmful diffusive species, the mechanism of antimicrobial activity is the deterioration of cytoplasmic proteins, such as ribosomes, by the incorporation of nanoparticles into the cytoplasm of bacteria. Also, positively-charged metallic nanoparticles exhibit antimicrobial properties by direct interactions with the cell membrane^17,18^. The antimicrobial mechanisms of these nanomaterials are related to the functional disorders of cell membrane or cytoplasmic proteins, rather than of cell walls, through which the nanomaterials can easily pass. On the other hand, nanostructured substrates, which are thin materials with nanostructures on their surfaces whose macroscopic side lengths are usually on the larger order than millimetres, have also been reported to exhibit antimicrobial properties^19–21^. It has been suggested that the damage to cell membranes by these nano-rough structures might occur through changes to the expression of genes related to the cell membrane^19^. The surface nanostructure influences their ability to affect the activity of bacteria^22^. Nanostructured substrates cannot pass through the cell wall because of their bulky dimensions, with typical macroscopic lengths larger than millimetres. Hence, their antimicrobial mechanisms are presumably related to disorder or denaturation of cell walls. However, there are no investigations from the biophysical viewpoint about the interactions between nanostructured surfaces and the cell wall, and the origin of the antimicrobial activity of nanostructured surfaces is unknown.

Recently, nanoporous Au (NPG), which has an open porous structure with pores and ligaments in the nanometre range^23^, was found to exhibit antimicrobial properties against *E*. *coli* and *S*. *epidermidis*^24^. In this case as well, its antimicrobial activity must be related to interactions between nanostructured surfaces and the cell wall, because NPG is too bulky to pass through cell walls of bacteria. In previous studies, the antimicrobial activity of NPG exhibited several peculiarities: (1) the source of the antimicrobial activity of NPG was neither metal ions nor ROS, which was clarified by inductively-coupled plasma atomic emission spectroscopy and Merckoquant peroxide test, (2) direct contact between bacteria and NPG substrate was necessary for the antimicrobial efficacy of NPG, because the antimicrobial activity of NPG was almost zero at high relative humidity (RH) (=90%), where the frequency of contact between bacteria and substrate was minimised, (3) the microarray analyses showed that NPG disturbed the function of the cell membrane of *E*. *coli*, and (4) the measurement of viable bacteria count showed that a long incubation time of 24 h was needed to cause the antimicrobial activity of NPG. From (2) and (3), the antimicrobial mechanism of NPG is inferred to be composed of two interactions: one between gold surface and cell wall, and the other between cell wall and cell membrane. Nanoporous metals have large lattice strains up to 10% at the surfaces, because the nanosized ligaments have high positive and negative curvatures^25–28^. The large lattice strains induce hyperpolarisation at the surface, as shown later. Hence, the antimicrobial activity of NPG may be a result of interactions related to the hyperpolarisation induced by NPG. Also, bacteria killed by NPG show leakage of cytoplasm^24^. This fact suggests that the strength of cell walls may be altered by interactions with NPG. Hence, in the present study, the interactions between NPG and *E*. *coli* are investigated from the viewpoint of hyperpolarisation and strength of the cell wall. In regard to the hyperpolarisation, fluorescent microscopic observation was used to study *E*. *coli* cultured on flat Au (FG) and NPG. In addition, molecular dynamics (MD) simulations and first-principles calculations were carried out to investigate the hyperpolarisation mechanisms of the cell wall. As mentioned above, it is important to account for the large tensile and compressive lattice strains up to 10% at the surface of nanoporous metals^25–28^. Thus, two Au cell models, one with 5% tensile lattice strain and the other with 5% compressive lattice strain, were used as models of NPG in the first-principles calculations. In regard to the strength of the cell wall, the elastic moduli of the cell walls of *E*. *coli* were experimentally measured by scanning probe microscopy (SPM), and the elastic modulus of peptidoglycan, a major component of the cell wall, was calculated by MD simulations. These experiments and simulations shed light on the interactions between Au surface and cell wall. Next, the effects of the hyperpolarisation on the lipid bilayer and potassium channel were investigated by MD simulations, providing insights into the interactions between the cell wall and cell membrane. As will be discussed, the hyperpolarisation plays a critical role in both interactions. Previously, MD simulations of the interactions between organic molecules and metallic surfaces have used empirical potentials such as CHARMM-METAL^29^. However, the empirical potentials cannot treat the effects of charge transfer and exchange interactions. Thus, in the present work, cooperation of MD simulations and first-principles calculations was used for investigating the hyperpolarisation. This computational strategy allows a deeper understanding of the role of hyperpolarisation in antimicrobial activity.

## Results and Discussion

**Figure 1:**
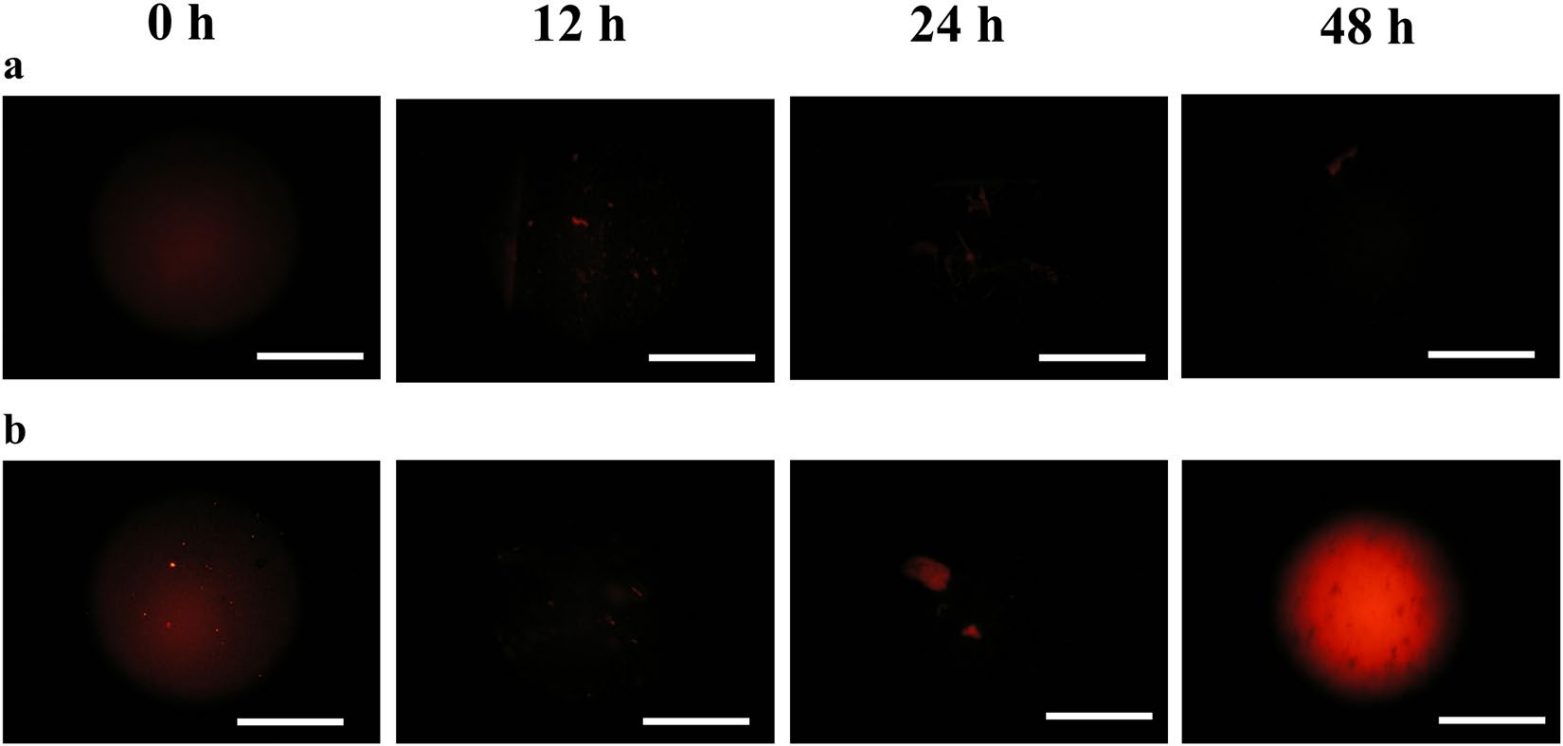
Fluorescent microscopic images of (**a**) *E*. *coli* cultured on nanoporous Au (NPG) and (**b**) *E*. *coli* cultured on flat Au (FG), where *E*. *coli* was cultured with a DiSC_3_(5) fluorescent probe. Scale bar = 200 µm. For *E*. *coli* on FG, only weak fluorescence is emitted during the incubation times of 0–24 h, but this emission becomes markedly more intense after 48 h of incubation. However, for *E*. *coli* on NPG, no fluorescence is observed after 48 h of incubation time. The fluorescent experiments were conducted 5 times and the same results were obtained each time.

Figure 1 shows fluorescent microscopic observations for *E*. *coli* cultured on NPG and on FG substrates. For *E*. *coli* cultured on FG, only weak fluorescence was emitted during the incubation times of 0–24 h, but the emission became markedly more intense after incubation for 48 h. For *E*. *coli* cultured on NPG, the fluorescence behaviour during the incubation times of 0–24 h was almost the same as that on FG. However, no fluorescence was observed after incubation for 48 h, unlike for *E*. *coli* on FG. The fluorescent experiments were conducted 5 times and the same results were obtained each time.

DiSC_3_(5)^30^ was used as the fluorescent probe in the fluorescent experiments. Dimeric DiSC_3_(5), which does not emit fluorescence, tends to be bound to negatively-polarised matter such as cell membrane, with a resting membrane potential, and dimeric DiSC_3_(5) decomposes into monomeric ones without polarized matters and monomeric DiSC_3_(5) emit fluorescence^30^. Hence, no fluorescence is emitted when the cell membrane has a certain membrane potential, while fluorescence is observed when this membrane potential is lost. The results in Fig. 1 show that *E*. *coli* was alive on FG for the first 24 h of incubation, which agrees with a previous experimental study of the viable bacterial count^24^; however, the membrane potential was lost, accompanied by the death of *E*. *coli*, after incubation for 48 h. The visual observation showed that the extinction of *E*. *coli* at 48 h was attributed to drying of the culture medium^24^. In the case of *E*. *coli* on NPG, it is noteworthy that no fluorescence was found even after 48-h incubation, despite the death of *E*. *coli*. This suggests that a certain part of each *E*. *coli* cell was hyperpolarised by NPG, and dimeric DiSC_3_(5) remained bound to the hyperpolarised part. The hyperpolarisation must be kept after the *E*. *coli* was dead.

**Figure 2:**
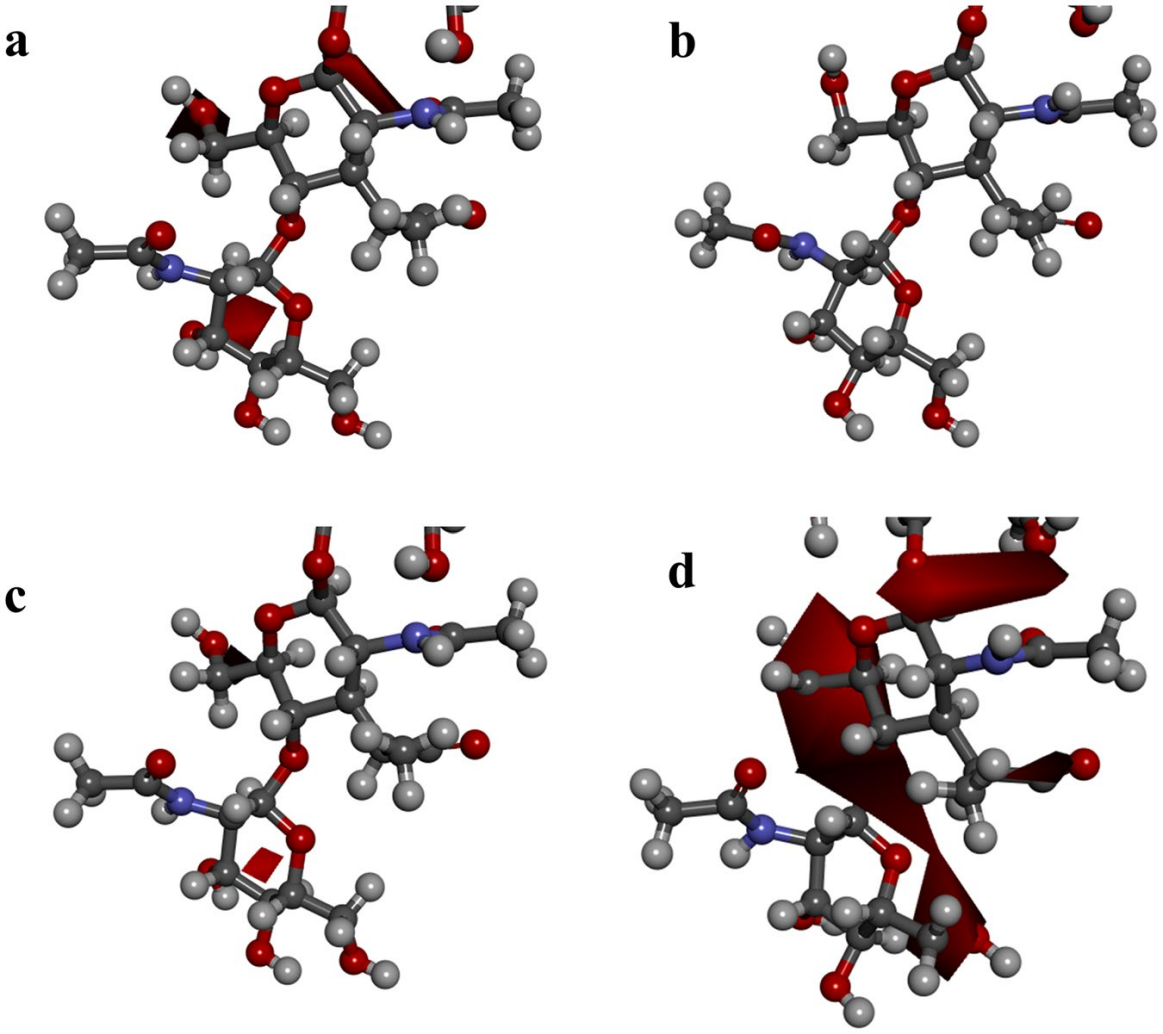
Electrostatic potentials of a part of peptidoglycan (glycan chain) close to the cell membrane, interacting with (**a**) no Au, (**b**) flat Au (FG), (**c**) nanoporous Au (NPG) (+5% strain) and (**d**) NPG (−5% strain), where the isosurface is plotted as the negative value of −20 kT. Only slight negative hyperpolarisation is observed for peptidoglycan interacting with no Au, FG or NPG (+5% strain). However, large negative hyperpolarisation is found for peptidoglycan interacting with NPG (−5% strain).

When *E*. *coli* is attached to the NPG surface, the contact site is the cell wall. The cell wall is composed of a network of peptidoglycan. Hence, peptidoglycan is a promising candidate for the material that undergoes hyperpolarisation in *E*. *coli*. To verify this hypothesis, the interactions of peptidoglycan with NPG were investigated by a combination of MD simulations and first-principles calculations. Figure 2 shows the electrostatic potentials of the glycan chain (a part of a peptidoglycan network) located close to a cell membrane, interacting with no Au substrates, FG (0% strain) or NPG (+5% and −5% strain). The isosurface is plotted as the negative value of −20 kT. Peptidoglycan interacting with NPG (−5% strain) showed a larger negative electrostatic potential than the non-interacting peptidoglycan, while no such difference was observed for peptidoglycans interacting with either FG or NPG (+5% strain). Clearly, the surface of peptidoglycan was negatively hyperpolarised by NPG. The area surrounding the surface of NPG showed a negative electrostatic potential under −5% strain, while this was not observed for the FG surface (Fig. S1). The vicinity of a metallic surface is generally characterised by an electric double layer, caused by the seeping of valence electrons out of the surface^31^, making the surface positively charged and the surrounding negatively charged. Here, the magnitude of the electric double layer was enhanced for NPG under −5% strain, as shown in Fig. S1. Therefore, the negative hyperpolarisation of peptidoglycan can probably be attributed to the enhanced positive charge at the NPG surface.

**Figure 3:**
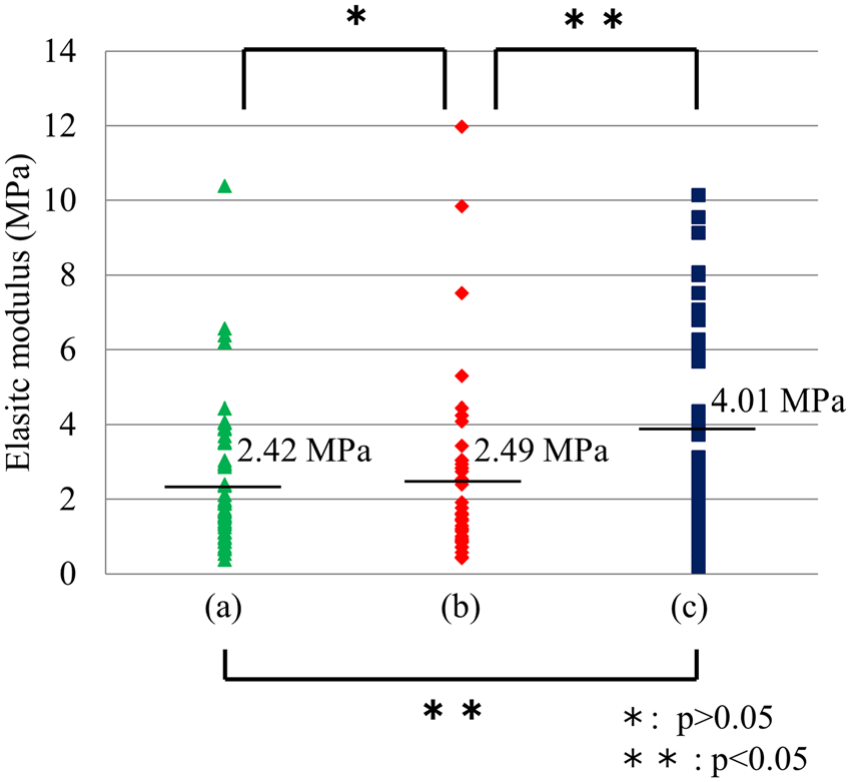
The elastic modulus of the cell wall, measured by scanning probe microscopy, of (**a**) raw *E*.*coli* (not cultured on a Au substrate), (**b**) *E*. *coli* cultured on flat Au (FG) and (**c**) *E*. *coli* cultured on nanoporous Au (NPG), where the bars show the average values. According to the *t*-test, there is no significant difference in elastic modulus between raw *E*. *coli* and *E*. *coli* cultured on FG; however, the elastic modulus of *E*. *coli* cultured on NPG is larger than the others.

In the previous study^24^, bacteria killed by NPG were found to leak cytoplasm. If the elastic modulus of peptidoglycan is weakened by the hyperpolarisation induced by NPG and the weakened peptidoglycan cannot stand the turgor pressure of a cell, the leakage of cytoplasm will occur. To verify this, the elastic modulus was investigated by the SPM measurements. Figure 3 shows the elastic moduli of raw *E*. *coli* (not cultured on a Au substrate), *E*. *coli* cultured on FG and *E*. *coli* cultured on NPG. The average elastic modulus was 2.42 MPa for raw *E*. *coli*, 2.49 MPa for *E*. *coli* cultured on FG and 4.01 MPa for *E*. *coli* cultured on NPG. Previous measurements of the elastic moduli of organisms such as *E*. *coli* have ranged from 0.01 to 800 MPa^32^, depending on measurement methods and conditions. The values obtained in the present investigation thus fall within this range. According to the *t*-test, there is no difference in elastic modulus between raw *E*. *coli* and *E*. *coli* incubated on FG; however, the elastic modulus of *E*. *coli* incubated on NPG is larger than the others. Thus, the interaction with NPG clearly strengthened the cell wall of *E*. *coli*. Previous studies also reported an increase in rigidity for malaria-infected red blood cells^33–35^.

**Figure 4:**
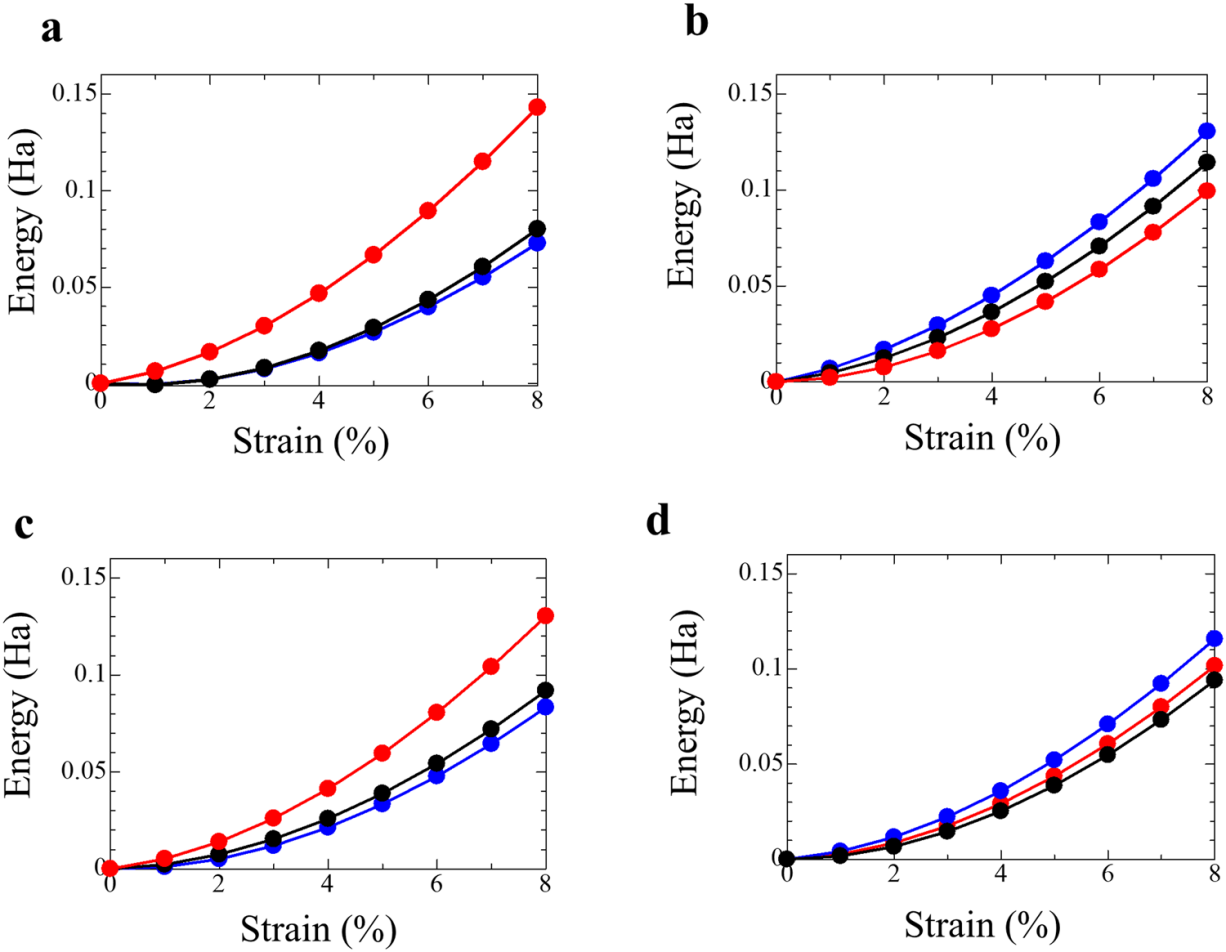
Energy–strain curves calculated by first-principles tensile tests for four parts of (**a**) glycan chain, where (**a**–**d**) correspond to the four parts (**a**–**d**) of a glycan chain shown in supplemental Figure S2. Blue, red and green lines show results of glycan chain interacting with no Au, flat Au (FG) or nanoporous Au (NPG) (−5% strain), respectively. The interaction energy of the glycan chain with NPG is greater than those with no Au and with FG in all four parts.

The increased elastic modulus of the cell wall of *E*. *coli* cultured on NPG was related to the hyperpolarisation of peptidoglycan. Here, first-principles calculations were carried out to investigate in detail the effects of the hyperpolarisation on the elastic modulus. The number of atoms that can be investigated by first-principles calculations is limited. Here, four parts of the glycan chain, shown by arrows in Fig. S2, were investigated by the first-principles tensile tests. The energy–strain curves for the four parts of the glycan chain are shown in Fig. 4. For *E*. *coli* on NPG, the energy as a function of the strain was higher than for raw *E*. *coli* and for *E*. *coli* cultured on FG in all four parts. This indicates that the elastic modulus of the peptidoglycan was increased by the interactions with NPG, which agrees with the experimental SPM measurements.

The relative locations of atoms of peptidoglycan were not changed by NPG (Fig. S3), however, the interatomic distances in peptidoglycan were changed by NPG. For example, the distance between two oxygen atoms in one part of the molecule, shown by arrows in Fig. S3, was 10.048 Å for raw *E*. *coli*, 10.526 Å for *E*. *coli* cultured on FG and 10.847 Å for *E*. *coli* cultured on NPG. The lengthening of the O–O distance for *E*. *coli* cultured on NPG is related to the negative hyperpolarisation induced by NPG. Thus, both the experimental and simulation results showed that the cell wall was strengthened by NPG, contrary to expectation. Therefore, the antimicrobial activity of NPG is unlikely to be a result of the weakening of the cell wall. The leakage of cytoplasm was observed for *E*. *coli* on NPG in the previous work^24^, suggesting that the leakage of cytoplasm occurred after the bacteria died. Recently, it was found that nanoprotrusions, whose height was about 500 nm, on the surface led to the mechanical rupture of bacteria^20,21^. In this case, the penetration of nanoprotrusions to 200 nm in depth inside bacteria was needed for causing the mechanical rupture of bacteria^20^. However, the pore or ligament size in NPG is about 20 nm, and therefore the mechanical rupture of bacteria cannot be caused by NPG.

Transcriptomic analyses^24^ have revealed that NPG disturbs the function of the cell membrane, suggesting that the hyperpolarisation of the cell wall affects the structure or function of the cell membrane. One of the main components of a cell membrane is the lipid bilayer. Hence, the interactions of the lipid bilayer with hyperpolarised peptidoglycan were investigated by MD simulations. The results showed that the lipid bilayer interacting with the hyperpolarised peptidoglycan did not undergo critical damage, relative to the lipid bilayer interacting with the non-hyperpolarised peptidoglycan (Fig. S4). Positively charged Au nanoparticles are known to cause critical damage to the cell membrane of bacteria^17,18^, which has also been verified by MD simulations^36,37^. In the present investigations, however, the cell wall was negatively charged, not positively, and the magnitude of this charge was much smaller than that of the positively charged Au nanoparticles. Thus, the hyperpolarised cell wall did not disrupt the lipid bilayer.

**Figure 5:**
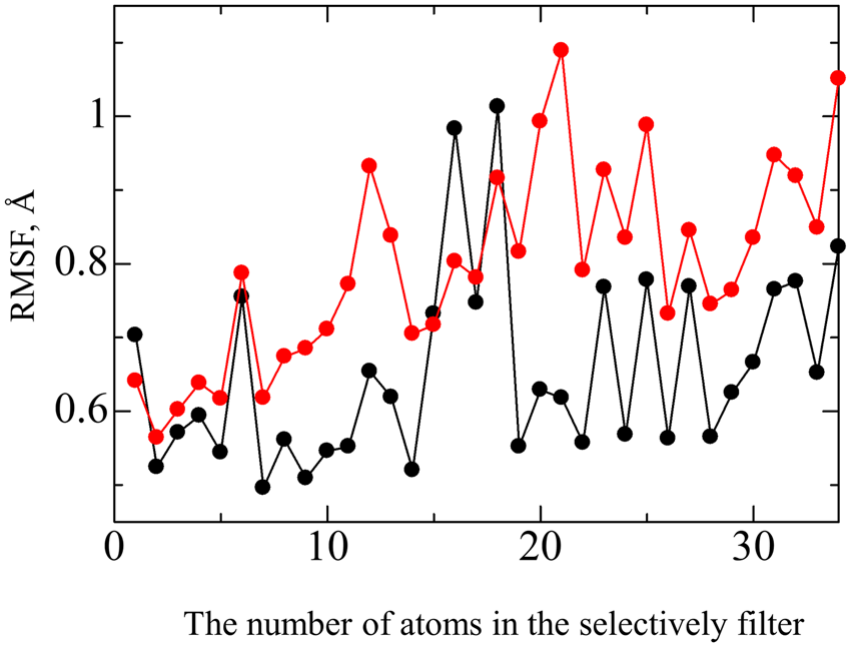
Variation in root-mean-square-fluctuation (RMSF) of atomic positions of the amino acids (Gly77, Tyr78 and Gly79) constituting the exit region of the selectivity filter, where the horizontal axis indicates the atoms in the amino acids and the atom numbering is shown in supplemental Table S1. Black and red lines show the RMSF value of the selectivity filter interacting with no Au and nanoporous Au (NPG), respectively. The RMSF values for the selectivity filter interacting with the hyperpolarised peptidoglycan are lower than those for the non-interacting selectivity filter, indicating that the structure of the selectivity filter fluctuates less in the presence of NPG.

The cell membrane consists not only of the lipid bilayer, but also of a variety of membrane proteins. Disruption of the lipid bilayer is not necessarily required for bacterial death. For example, in one study Au nanoparticles killed bacteria by inhibiting the activity of ATPase^11^. In the present work, the potassium channel was investigated because it plays a critical role in maintaining the ion concentration inside a cell, which controls a wide variety of cell functions. Hence, in this work, the interactions of the potassium channel with the hyperpolarised peptidoglycan were investigated by MD simulations. The results showed that the overall structure of the potassium channel was only slightly affected by the hyperpolarised peptidoglycan (Fig. S5), as in the case of the lipid bilayer. However, the hyperpolarised peptidoglycan did cause delicate differences in the atomic positions of the ion channel. Potassium ions pass through this channel 10,000 times faster than sodium ions, despite the close similarity in size of the two species. The high selectivity of the potassium channel is related to the sophisticated structure of the selectivity filter, which forms the most constricted part of the conduction pathway. If the structure of the selectivity filter is changed even slightly, critical damage will be caused to the functions of the ion channel. Figure 5 shows the variation in root-mean-square-fluctuation (RMSF) of the atomic positions of the amino acids (Gly77, Tyr78 and Gly79) constituting the exit region of the selectivity filter, where the horizontal axis indicates the atoms in these amino acids and the atom numbering is shown in supplemental Table S1. The RMSF is given by

1 $${\rm{RMSF}}=\sqrt{\frac{1}{t}}{\Sigma }{({x}_{i}({t}_{j})-{x}_{i})}^{2}$$

where *t*_*j*_ is the simulation time, *x*_*i*_(*t*_*j*_) is the position of atom *i* at simulation time *t*_*j*_, and *x*_*i*_ is the time-averaged position of atom *i*. The average RMSF was 0.68 Å for the selectivity filter interacting with the hyperpolarised peptidoglycan and 0.85 Å for the non-interacting selectivity filter, indicating that the structure of the selectivity filter fluctuated less in the presence of NPG. This difference of the average RMSF values has serious implications for the viability of the cells, being almost as large as the difference between a sodium ion (=1.02 Å) and a potassium ion (=1.38 Å). Any structural change to the selectivity filter strongly affects the velocity of potassium ions through the ion channel. Hence, any change in the structural fluctuation of the selectivity filter will affect the ion concentration inside a cell, and in turn a wide variety of cellular functions, irrespective of whether the velocity of potassium ions is increased or decreased by the reduction in fluctuation. A long incubation time of 24 h was needed for the antimicrobial activity of NPG^24^. This is quite different from the antimicrobial activity of metallic ions, which kill bacteria immediately after commencing incubation. Metallic ions immediately destroy the cell membrane. In contrast, the hyperpolarised peptidoglycan did not destroy or even damage the cell membrane, but rather caused delicate changes to the structure of the ion channel. Therefore, a longer time was needed for the bactericidal activity of NPG to be exerted.

## Conclusions

In conclusion, fluorescent microscopic observation, SPM measurements, and the cooperation of MD simulations and first-principles calculations were performed to investigate the antimicrobial mechanisms of NPG. The cell wall of *E*. *coli* was strengthened by NPG, indicating that the antimicrobial activity of NPG is not a result of the weakening of the cell wall. The cell wall of bacteria was negatively hyperpolarised by NPG, and the hyperpolarised cell wall caused delicate changes to the structure of the ion channel. Therefore, it is suggested that the hyperpolarisation induced by NPG plays a critical role in its antimicrobial activity.

## Methods

### Preparation of nanoporous and FG substrates

Au (>99.9 mass%) was sputtered on a glass slide (50 mm square and 1.2 mm in thickness) by radio-frequency (RF) magnetron sputtering (SVC-700RF, Sanyu Electron Co., Ltd., Tokyo, Japan). The Au layer was 90 nm in thickness, which is necessary to avoid delamination and fragmentation of NPG during dealloying because of its brittleness and shrinkage^23,25^. Then, a Au_0.3_Ag_0.7_ alloy with a thickness of 80 nm was sputtered on the Au thin film on the glass slide by RF magnetron sputtering. NPG was synthesised by dealloying of the Au-Ag/Au/glass slide (free corrosion) at 253 K for 24 h in 69 mass% HNO_3_, followed by thorough water washing. The surface structure of the fabricated NPG was observed by a scanning electron microscope (SEM, SU-6600, Hitachi High-Technologies). The quantitative chemical compositions of the samples were analysed by energy-dispersive x-ray spectroscopy (EDXS) equipped with the SEM. For comparison, Au (90 nm in thickness) on a glass slide was also prepared by RF magnetron sputtering as a bulk material without a nanoporous structure, and is referred to as FG. The SEM images and EDXS results are summarised in a previous publication^24^.

### Bacterial strain

Type strains of *E*. *coli* (K-12, NBRC 3301) were obtained from the National Institute of Technology and Evaluation (Tokyo, Japan). We incubated the bacteria in Luria–Bertani (LB) medium at 308 K for 48 h before the following experiments. Casein-peptone glucose yeast extract agar (Wako Pure Chemical Industries Ltd., Osaka, Japan) was used for the incubation.

### Fluorescent microscopic observation

400 µL of culturing suspension (1/500 nutrient broth in 100 mmol/L KCl) containing 0.4 µmol/L of DiSC_3_(5) (Tokyo Chemical Industry Corporation) and 1 × 10^7^ colony forming unit (CFU)/mL of *E*. *coli* was dropped onto the nanoporous and FG substrates. The *E*. *coli* on nanoporous and FG substrates was cultured at 308 K for 48 h at RH = 50%. The fluorescence at wavelengths of 649–670 nm was observed at given incubation times using a fluorescent optical microscope (BX53, Olympus, Tokyo, Japan).

### Preparation for SPM measurements

One quantity of platinum ear of bacteria incubated was put into 5 mL of 1/500 nutrient broth, followed by vortex mixing. 400 µL of bacterial suspension was dripped onto the samples, and the bacterial suspensions were then covered with polyethylene film. The bacterial suspensions were incubated on the samples for a given period (up to 24 h) in humidity-controlled incubators at 35 °C. The incubated bacteria were completely recovered on the samples by 5 mL of phosphate-buffered saline. We used these suspensions for the following measurements.

To immobilise the bacterial cells on the glass substrate, cover glasses were coated with poly-l-lysine (Cultrex)^38,39^. The cover glasses were covered with 150 mL of 0.02 mass% poly-l-lysine aqueous solution for 30 min and rinsed with water. The cover glasses were then air-dried for 2 h. 200 µL of the bacterial suspension was deposited onto a cover glass coated with poly-l-lysine. After 20 min, the bacterium-coated glass substrate was then gently rinsed with water three times to remove excess and loosely attached bacterial cells. The cover glass with immobilised bacteria was then transferred into the SPM fluid cell for imaging and force-measurements in water.

### Measurements of elastic modulus with SPM

SPM measurements were performed using an SPM-9700 (Shimadzu) on the above-mentioned bacterial cells immobilised on glass substrates coated with poly-l-lysine and placed in the SPM fluid cell. Silicon nitride cantilevers with a nominal spring constant of 0.15 N m^−1^ were purchased from Olympus (OMCL-TR800PSA-1). To obtain force curves, we used force-mapping in contact mode, where the tip of the cantilever was indented at 50 nm s^−1^ above the centre of *E*. *coli*. The elastic modulus was then calculated using Johnson–Kendall–Roberts (JKR) theory^40,41^. The indentation was conducted at least 35 times.

### Computational details

Previously, MD simulations of the interactions between organic molecules and metallic surfaces have used empirical potentials such as CHARMM-METAL^29^. However, MD simulations using empirical potentials cannot treat the effects of charge transfer and exchange interactions. On the other hand, first-principles calculations can treat the effects of charge transfer and exchange interactions^42^, but the number of atoms in the system is very limited. Hence, a combination of MD simulations and first-principles calculations was performed to investigate the interactions between the Au surface and peptidoglycan. In addition, another MD simulation was carried out to investigate the interactions between peptidoglycan and the membrane systems. Details of the computational methods are described in the supplemental information.

## Electronic supplementary material

Supplemental Information
