# Supplementary material for: Antimicrobial mechanisms due to hyperpolarisation induced by nanoporous Au
Source: Sci Rep. 2018 Mar 1;8:3870. doi: 10.1038/s41598-018-22261-5 (PMC5832825; doi:10.1038/s41598-018-22261-5)
Supplement: Supplementary file 1 — Supplemental Information [file 41598_2018_22261_MOESM1_ESM.pdf]

Suppplemental Information for Antimicrobial mechanisms due to hyperpolarization induced by nanoporous Au

Naoki Miyazawa*, Masataka Hakamada and Mamoru Mabuchi

Graduate School of Energy Science, Kyoto University,

Yoshidahonmachi, Sakyo, Kyoto 606-8501, Japan

Computational details

Construction of peptidoglycan model

A bacterial cell wall consists of a network of peptidoglycan. The structural characteristics of peptidoglycan have been studied by electron microscopy[1] and molecular dynamics (MD) simulations[2,3]. Peptidoglycan is composed of repeating units consisting of a disaccharide (*i.e.*, N-acetylglucosamine (GlcNAc) and N-acetylmuramic acid (MurNAc)) and a cross-link peptide. In the present study, repeating units of a stem (L-Ala-D-iso-Gln-L-Lys-D-Ala-D-Ala) and a bridge (Gly1- Gly2- Gly3 – Gly4 – Gly5) were selected as a component of cross-link peptide in accordance with the previous study[4].

The 3-dimensional structure of peptidoglycan is still not known although many studies tried to clarify the architecture of peptidoglycan[2,3]. Two major candidates of peptidoglycan structure have been proposed: the layered model[5] and the scaffold model[2,3]. It has been shown that the scaffold model well represents the mechanical properties of cell wall, compared with the layered model[2,3]. Thus, the scaffold model was used in the present study.

The components (*i.e.*, a disaccharide and a cross-link peptide) of peptidoglycan were constructed and the geometry optimizations were performed using the Gaussian program

package. Four GlcNAc-MurNAc disaccharides were connected each other and the pentapeptides and the pentaglycine were attached to the glycan chains to construct the cross-link structure. Thereafter, a terminal of pentapeptides was connected to another GlcNAc-MurNAc strand, and then MurNAc molecule, which was not still connected to the cross-link structure in GlcNAc-MurNAc strand, was connected to other pentapeptides and pentaglycine. By repeating this process, a scaffold model of peptidoglycan was constructed.

Energy minimizations and MD calculations were performed to obtain a stabilized structure of peptidoglycan by the Discovery studio 4.0 software (Biovia Inc, San Diego, CA), using the CHARMM forcefield[6]. The peptidoglycan was immersed in a spherical water solvation. The center of water solvent was positioned at the mass center of peptidoglycan and the diameter of spherical solvent water was 50.0 nm. Counter ions of 43 Na^{+} and 43 Cl^{-} were added to neutralize the system. The system was energy-minimized using the steepest decent algorism (200,000 steps) and the conjugate gradient algorism (100,000 steps). MD simulations were performed with the time step of 2.0 fs. The system was gradually heated from 5 to 300K for 4ps to activate thermal motion in the system. The system was equilibrated for 1ns to obtain a stable structure of peptidoglycan with the constant number of particles, volume and temperature (NVT) ensemble. Finally, the 10ns

NVT simulations were performed. The peptidoglycan model obtained by the MD simulations is shown in Supplemental Fig. S2.

Interactions between nanoporous Au and peptidoglycan

An interaction between MurNAc, which is a part of peptidoglycan, and a surface of flat Au (FG) or nanoporous Au (NPG) was investigated by first principles calculations. A (111) FG model with a surface unit cell of $4\sqrt{3} \times 3\sqrt{3}$ was used, where the lattice strain of the FG model was 0%. The model cell consisted of four (111) Au layers and contained 144 Au atoms. The vacuum gap of 15 Å was added to create the surface. In NPG, large lattice strains exist at its surface. Thus, a lattice strain of +5% or -5% was loaded into the (111) layers of the FG model and two NPG models were constructed: NPG (+5% strain) and NPG (-5% strain).

The geometry optimization calculations were performed on the FG and the NPG models by first principles calculations using the Dmol3 code[7,8]. In the DMol3 method, the physical wave functions were expanded in terms of the accurate numerical basis sets. The exchange-correlation energies were treated according to the generalized gradient approximation (GGA) with the Perdew-Wang 1991 (PW91) approximation[9] to deal with the core (DNP). The ultrasoft pseudopotentials[10] represented in reciprocal space were

used for all elements in the calculations. Optical Bloch equation (OBE) calculations were used to set the van der Waals interactions into calculations. A Fermi smearing of 0.005 hartree (1 hartree = 27.2114 eV) was adopted. The tolerances of energy, gradient, and displacement convergence were 1.0×10^{-5} hartree, 2.0×10^{-3} hartree/Å, and 5×10^{-3} Å, respectively. The Brillouin zone of 2×2×1 using a Monkhorst–Pack k-point mesh[11] was used. The geometry optimization calculations were carried out with spin polarization. The bottom two layers of the FG and the NPG models were frozen during geometry optimization calculations. The Au surface was assumed to make contacts with the front edge of a disaccharide because glycan chains stood vertically on the surface of cell membrane in the scaffold model. Thus, the MurNAc, which is located at the edge of a glycan chain, was putted on the FG and the two NPG models (+5% and -5% strains).

MurNAc was positioned on fcc, hcp, atop and bridge sites of the FG model and the geometry optimization calculations were performed on the four sites using the first principles calculations. The lowest value of adsorption energy was obtained in the case of the atop site. Thus, the position of MurNAc was determined to be the atop site for all the models.

After the geometry optimizations of MurNAc molecule on the atop site for the FG and the NPG models, each MurNAc was put back at the same position in the original

peptidoglycan model and 1 ns MD simulations were performed again, where the atomic positions of MurNAc molecule were not relaxed during the calculations.

Electrostatic potentials of peptidoglycan

After the MD simulations about peptidoglycan, the electrostatic potentials of obtained peptidoglycan were calculated by solving the Poisson Boltzmann equation using the finite difference method implemented in Delphi program[12,13] with the Discovery studio 4.0. The values of atomic radii and partial atomic charges were taken from the CHARMM parameter set. The peptidoglycan was divided into a three-dimensional cubical grid and the electrostatic potential at each grid point was computed. Electrostatic potentials of a part of peptidoglycan close to cell membrane are shown in Fig. 2.

First principles tensile tests

First principles tensile tests with the Dmol3 code[7,8] were performed to investigate effects of the hyperpolarization of peptidoglycan on the elastic modulus. Four parts of peptidoglycan shown by arrows in Fig. S2, which were absorbed on NPG, were investigated. The distance between two oxygen atoms shown by arrows in Fig. S3 was increased by 1% strain without relaxation. This operation was repeated to 8% strain. The

exchange-correlation energies were treated according to the generalized gradient approximation (GGA) with the Perdew-Wang 1991 (PW91) approximation[9] to deal with the core (DNP). The ultrasoft pseudopotentials[10] represented in reciprocal space were used for all elements in the calculations. Optical Bloch equation (OBE) calculations were used to set the van der Waals interactions into calculations. A Fermi smearing of 0.005 hartree (1 hartree = 27.2114 eV) was adopted. The tolerances of energy, gradient, and displacement convergence were 1.0×10^{-5} hartree. The energy calculations were carried out with spin polarization.

Lipid membrane interacting with hyperpolarized cell wall

The 24 POPE lipid were solvated in 1,412 water molecules using CHARMM-GUI web site. A part of peptidoglycan interacting with FG or NPG was positioned on lipid membrane (Fig. S4). Periodic boundary conditions were applied. MD simulations were performed using the CHARMM force field[6] with gromacs 5.1.1 code[14,15]. The position of peptidoglycan was fixed during the simulations. The system was energy-minimized using the steepest decent algorism (500,000 steps). MD simulations were performed with the time step of 1.0 fs. The system was equilibrated for 10 ns to obtain a stable structure of

peptidoglycan with the constant number of particles, volume and temperature (NVT) ensemble. Finally, the 100 ns NVT simulations were performed.

Potassium channel interacting with hyperpolarized cell wall

Initial coordinates for potassium channel were taken from the crystal structures 1K4C. The channel was embedded in a bilayer of 2,182 POPE lipid and solvated in 15,078 water molecules and 15 Cl^- ions using CHARMM-GUI web site. A part of peptidoglycan interacting with FG or NPG was positioned on the ion channel (Fig. S5). Periodic boundary conditions were applied. MD simulations were performed using the CHARMM force field[6] with gromacs 5.1.1 code[14,15]. The position of peptidoglycan was fixed during the simulations. The system was energy-minimized using the steepest decent algorism (500,000steps). MD simulations were performed with the time step of 1.0 fs. The system was equilibrated for 10 ns to obtain a stable structure of peptidoglycan with the constant number of particles, volume and temperature (NVT) ensemble. Finally, the 100 ns NVT simulations were performed.

References

1. Dubochet, J., McDowall, A. W., Menge, B., Schmid, E. N. & Lickfeld, K. G. Electron microscopy of frozen-hydrated bacteria. *J. Bacteriol.* **155**, 381-390 (1983).

2. Meroueh, S. O., Bencze, K. Z., Hesek, D., Lee, M., Fisher, J. F., Stemmler, T. L. & Mobashery, S. Three-dimentional structure of the bacterial cell wall peptidoglycan. *PNAS* **103**, 4404-4409 (2006).

3. Dmitriev, B., Toukach, F. & Ehlers, S. Towards a comprehensive view of the bacterial cell wall. *Trends in Microbiol.* **13**, 569-574 (2005).

4. Yusupov, M., Bogaerts, A., Huygh, S., Snoeckx, R., van Duin, A. C. T. & Erik, N. C. Plasma-induced destruction of bacterial cell wall components: a reactive molecular dynamics simulation. *J. Phys. Chem. C* **117**, 5993-5998 (2013).

5. Ghuysen, J. M. Use of bacteriolytic enzymes in determination of wall structure and their role in cell metabolism. *Bacteriological reviews* **32**, 425-464 (1968).

6. MacKerell, A. D. Jr. et al. All-atom empirical potential for molecular modeling and dynamics studies of proteins. *J. Phys. Chem. B* **102**, 3586-3616 (1998).

7. Delley, B. J. An all-electron numerical method for solving the local density functional for polyatomic molecules. *J. Chem. Phys.* **92**, 508-517 (1990).

8. Delley, B. J. From molecules to solids with the $Dmol^3$ approach. *J. Chem. Phys.* **113**,

7756-7764 (2000).

9. Perdew, J. P. et al. Atoms, molecules, solids, and surfaces: Application of the generalized approximation for exchange and correlation. *Phys. Rev. B* **46**, 6671-6687 (1992).

10. Vanderbilt, D. Soft self-consistent pseudopotentials in a generalized eigenvalue formalism. *Phys. Rev. B* **14**, 7892-7895 (1990).

11. Monkhorst, H. J. & Pack, J. D. Special points for Brillouin-zone integrations. *Phys. Rev. B* **13**, 5188-5192 (1976).

12. Rocchia, W., Alexov, E. & Honig, B. Extending the applicability of the nonlinear Poisson-Boltzmann equation: multiple dielectric constants and multivalent ions. *J. Phys. Chem. B* **105**, 6507-6514 (2001).

13. Rocchia, W., Sridharan, S., Nicholls, A., Alexov, E., Chiabrera, A. & Honig, B. Rapid grid-based construction of the molecular surface and the use of induced surface charge to calculate reaction field energies: applications to the molecular systems and geometric objects. *J. Comput. Chem.* **23**, 128-137 (2002).

14. Spoel, D. V. D., Lindahl, E., Hess, B., Groenhof, G., Mark, A. E. & Berendsen, H. J. C. GROMACS: fast, flexible, and free. *J. Comput. Chem.* **26**, 1701-1718 (2005).

15. Abraham, M. J., Murtola, T., Schulz, R., Pall, S., Smith, J. C., Hess, B. & Lindahl, E.

GROMACS: High performance molecular simulations through multi-level parallelism from laptops to supercomputers. *Software X* **1-2**, 19-25 (2015).

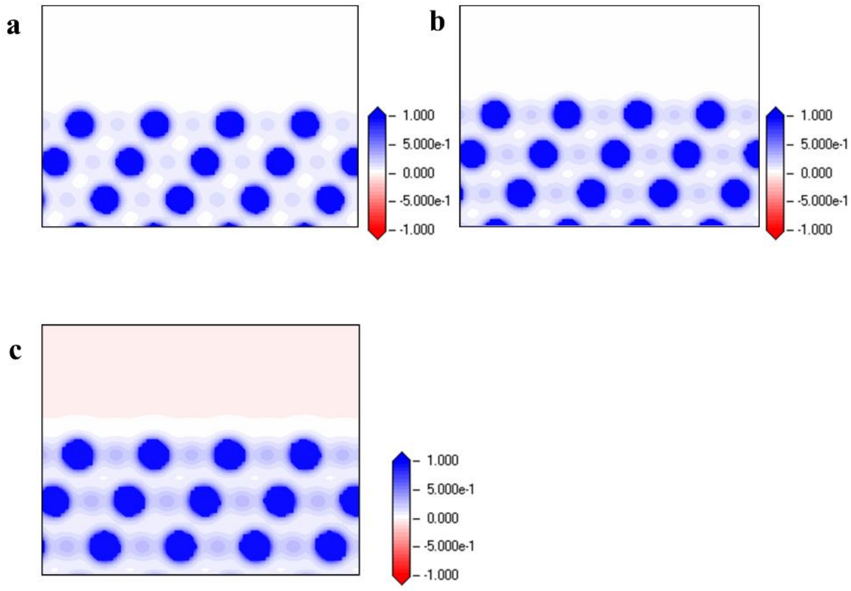

Figure S1. Electrostatic potential for (a) flat Au (FG), (b) nanoporous Au (NPG) (+5% strain) and (c) NPG (-5% strain). The electrostatic potential is negative at a region outside the surface of NPG (-5% strain), comparing with FG, indicating that the negative strain enhances the positive charge at the surface of NPG.

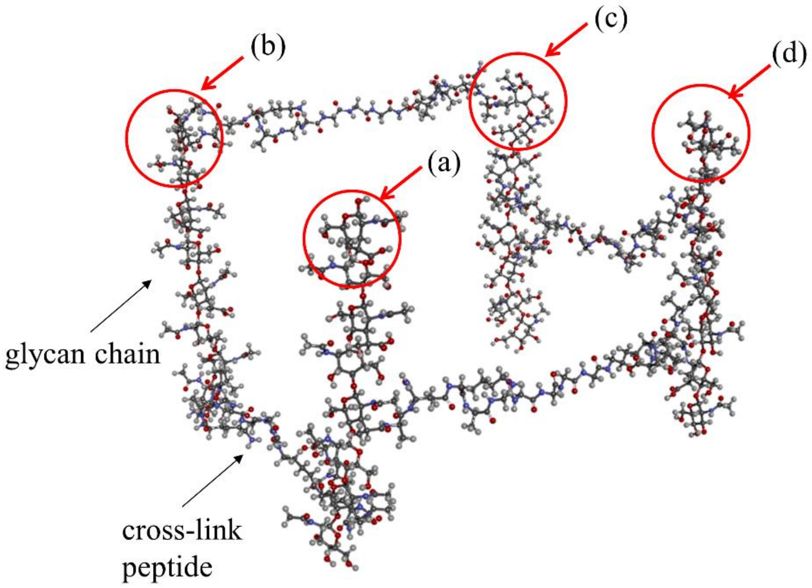

Figure S2. A peptidoglycan model after stability computation with 10ns NVT calculation. Gray, red, blue and white spheres show carbon, oxygen, nitrogen and hydrogen atoms, respectively. Four glycan chains are connected by cross-link peptide. Red circles (a)-(d) indicate parts of peptidoglycan calculated by first principles tensile tests (Fig. 4). The parts are the one adsorbed on NPG.

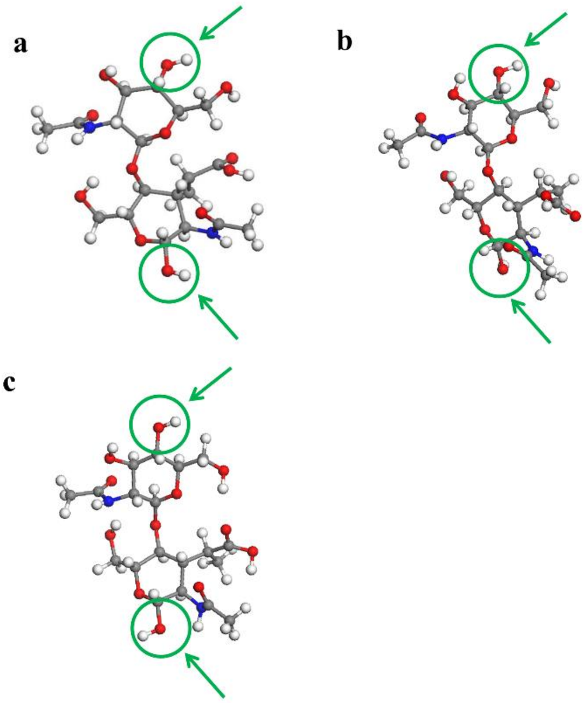

Figure S3. Simulation models of a part of glycan chain for first-principle tensile tests, (a) glycan chain interacting with no Au, (b) glycan chain interacting with nanoporous Au (NPG) (+5% strain) and (c) glycan chain interacting with NPG (-5% strain). The part of glycan chain corresponds to the one shown by an arrow (a) in Fig. S2. Gray, red, blue and white spheres show carbon, oxygen, nitrogen and hydrogen atoms, respectively.

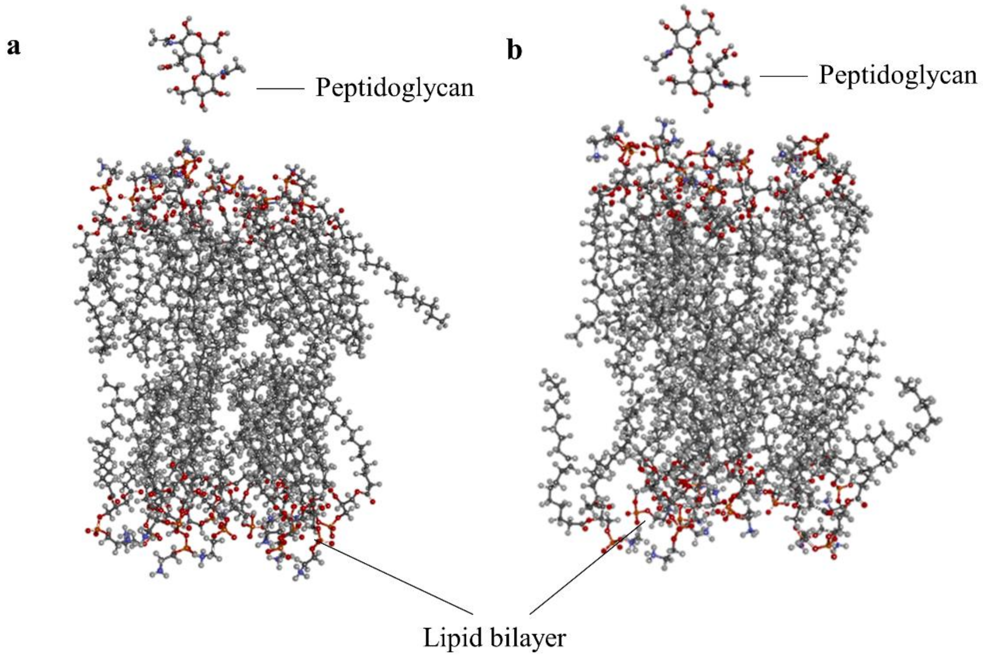

Figure S4. Lipid bilayer models interacting with peptidoglycan located on (a) flat Au and (b) nanoporous Au (-5% strain). No critical damage is found in lipid bilayer interacting with the peptidoglycan located on nanoporous Au.

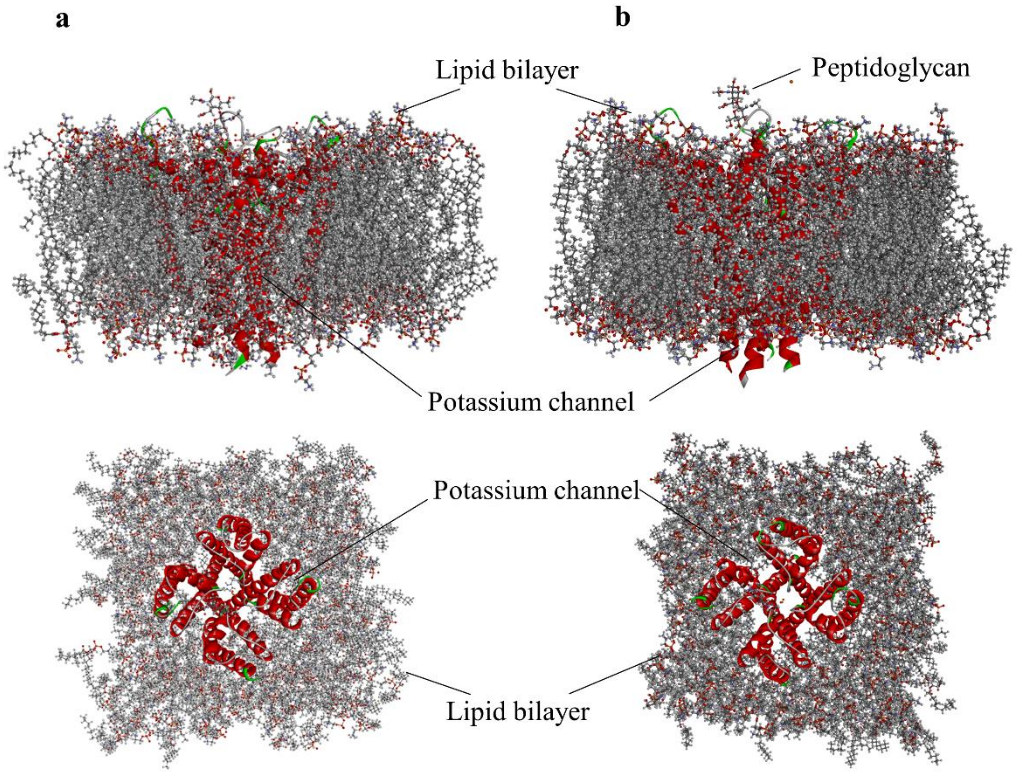

Figure S5. Schematic illustrations from side view (upper panel) and top view (lower panel) of potassium ion channel interacting with peptidoglycan located on (a) flat Au and (b) nanoporous Au (-5% strain). The whole structure of potassium channel is not affected by peptidoglycan located on nanoporous Au (-5% strain).

Supplemental Table S1 The number of atoms in Gly77, Tyr78 and Gly79. The schematic illustrations of amino acids and the descriptions of kind of atoms are shown in the right column, where gray, red, blue and white spheres show carbon, oxygen, nitrogen and hydrogen atoms, respectively.

Number	Atom	Amino acid
1	N	Gly77<br>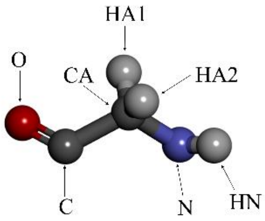
2	HN	
3	CA	
4	HA1	
5	HA2	
6	C	
7	O	
8	N	Tyr78<br>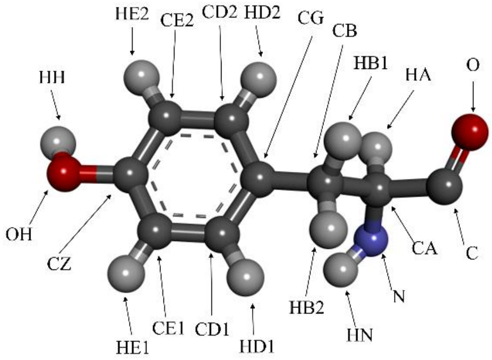
9	HN	
10	CA	
11	HA	
12	CB	
13	HB1	
14	HB2	
15	CG	
16	CD1	
17	HD1	
18	CE1	
19	HE1	
20	CZ	
21	OH	
22	HH	
23	CD2	
24	HD2	

25	CE2	
26	HE2	
27	C	
28	O	
29	N	Gly79<br>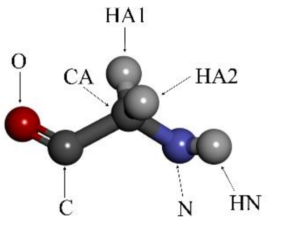
30	HN	
31	CA	
32	HA1	
33	HA2	
34	C	
35	O	
